# Interprofessional palliative care education for pediatric oncology clinicians: an evidence-based practice review

**DOI:** 10.1186/s13104-018-3905-5

**Published:** 2018-11-07

**Authors:** Sarah B. Green, Adelais Markaki

**Affiliations:** 1 School of Nursing, University of Alabama at Birmingham, 1720 2nd Ave. South, Birmingham, AL 35294-1210 USA; 2 Children’s Hospital Los Angeles, 4650 Sunset Blvd. #54, Los Angeles, CA 90027-6062 USA

**Keywords:** Palliative care, Interprofessional education, Pediatric oncology, Evidence-based practice

## Abstract

**Objective:**

Clinician education and expertise in palliative care varies widely across pediatric oncology programs. The purpose of this evidence-based practice review was to identify interprofessional palliative care education models applicable to pediatric oncology settings as well as methods for evaluating their impact on clinical practice.

**Results:**

Based on a literature search in PubMed, CINAHL and Embase, which identified 13 articles meeting inclusion/exclusion criteria, the following three themes emerged: (1) establishment of effective modalities and teaching strategies, (2) development of an interprofessional palliative care curriculum, and (3) program evaluation to assess impact on providers’ self-perceived comfort in delivering palliative care and patient/family perceptions of care received. Remarkably, health professionals reported receiving limited palliative care training, with little evidence of systematic evaluation of practice changes following training completion. Improving palliative care delivery was linked to the development and integration of an interprofessional palliative care curriculum. Suggested evaluation strategies included: (1) eliciting patient and family feedback, (2) standardizing care delivery measures, and (3) evaluating outcomes of care.

## Introduction

Children with cancer are a vulnerable population with complex needs. Despite advancements in treatments, cancer remains the fourth leading cause of death among children with up to 20% dying of their disease, and an even larger percentage experiencing physical and psychosocial suffering during and after treatment [1–4]. Early integration of palliative services into oncology care has been shown to reduce symptoms and suffering as well as to improve outcomes by providing meaningful person-and-family-centered care experiences [3, 5]. It also reduces healthcare costs by decreasing hospitalization days and emergency department visits [3, 6, 7]. An integrated model for delivering palliative care (PC) throughout the continuum, starting at diagnosis, has been recommended [8]. This interprofessional approach requires highly skilled team members working together to meet the needs of the pediatric oncology patient and family [9].

Despite growing evidence about the benefits of PC in pediatric oncology, clinician education and expertise varies widely across programs. Differential, and often suboptimal access to services is a rising concern [3]. A staggering 89% of parents reported their child experienced significant suffering at end-of-life and that symptoms were successfully controlled less than 30% of the time [4]. In a 2013 survey of pediatric palliative care programs, 69% of institutions reported having a PC team [1]. Yet, the majority of pediatric oncologists (75%) reported lacking formal training in end-of-life care [10]. This discrepancy suggests that PC expertise is concentrated in tertiary institutions with highly specialized teams, while oncology practices are lagging behind. To address this disparity, the National Consensus Project [9] issued guidelines calling for: (1) increasing the number of clinicians trained in interprofessional pediatric palliative care, and (2) expanding access to comprehensive PC teams throughout the continuum of care. Incorporating interprofessional education (IPE), training, and research for pediatric oncology providers is critical to improving quality of life for this population [2, 11, 12]. This evidence-based practice literature review aimed to identify potential knowledge gaps in regards to interprofessional palliative care education models, as well as methods for evaluating their impact on pediatric oncology practice.

## Main text

### Methods

The World Health Organization (WHO) Framework for Action on Interprofessional Education and Collaborative Practice definition for IPE was adopted [13]. For collaborative practice, the terms ‘interdisciplinary’ and ‘multidisciplinary’, often used interchangeably, were operationalized as follows: ‘interdisciplinary’ describes two or more academic disciplines working together to achieve a shared goal whereas, ‘multidisciplinary’ draws on the knowledge of different disciplines to work on a common problem with limited integrated interaction [14].

#### Search strategy and data analysis

**Fig. 1:**
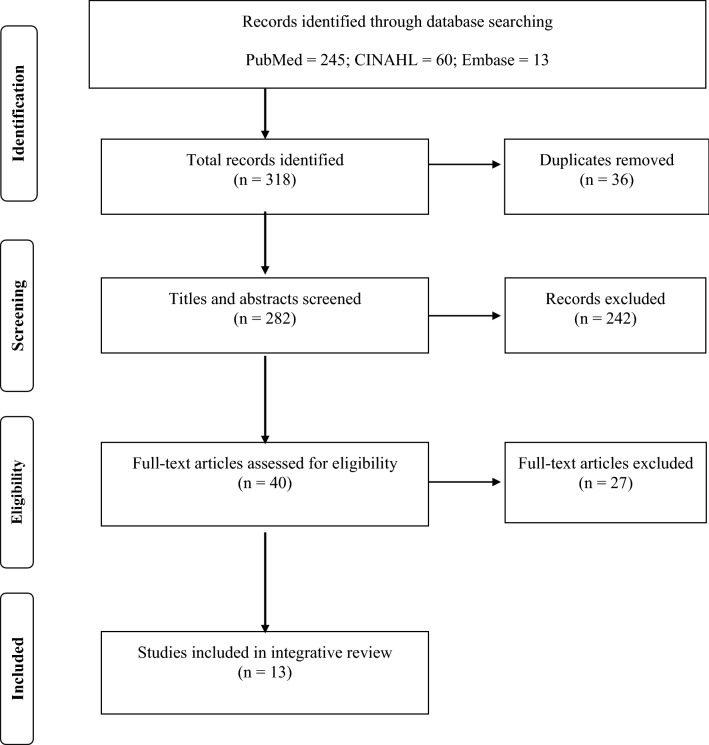
Literature search flow chart

A qualitative evidence synthesis was performed in PubMed, CINAHL, and Embase using the key search terms “*education, interprofessional education, palliative care, multidisciplinary team, oncology, AND pediatrics*.” (see Fig. 1). Only full-text, peer reviewed research articles published in English within the past 5 years (January, 2012–October, 2017) were included. Articles on interprofessional education models pertaining to the adult, non-oncologic population were selected to reflect that core PC competencies cross the age spectrum of populations [9]. Exclusion criteria were based on absence of (1) clinician education focus, (2) an interprofessional or multidisciplinary approach, and (3) an educational intervention. A total of 13 articles met all criteria, including 12 articles at level VI evidence and 1 at a level I.

Thematic analysis of the literature sample is reported in Table 1 with themes derived from the WHO educator and curricular mechanisms, including training, program content, learning outcomes, and shared-decision-making processes.

**Table 1:** Literature sample thematic analysis

Author (year)	Country	Design	Setting/sample	Discipline/roles	Relevant findings	Theme(s)^a^
Head et al. (2014) [15]	US	Pre–post-mixed-methods design	A large public metropolitan university, students participating in an interdisciplinary palliative oncology curriculum	Medical, nursing, social work, and chaplaincy students	Successful interdisciplinary palliative care education requires formative feedback, compromise, and focus on desired learning outcomes Content included case-based didactic modules, clinical rotations, and clinical reflective writing	#2
Head et al. (2016) [21]	US	Pre–post-mixed-methods design	373 students at a large public metropolitan university, students participating in an interdisciplinary palliative oncology curriculum	Medical, nursing, social work, and chaplaincy students	Quantitative evaluation revealed a significant improvement in student’s palliative care knowledge, skills, and interprofessional education readiness Qualitative results showed that experiential learning components including clinical shadow experiences and simulation with fellow students were reported as most valuable	#3
Hedlund (2013) [23]	US	Descriptive pilot study	A large community-based oncology practice, three pilot sites selected	Nurses, physicians, medical assistants, non-clinical staff	Fifty-four percent of nurses surveyed reported having increased confidence in their end of life skills Following initiation of program only three percent of patients discussed at clinical case conferences received chemotherapy in last 14 days of life Educational for clinical and non-clinical staff improved awareness and confidence in caring for patient with advanced cancer Staff reported improved communication and empathy across professions	#2, #3
Henderson et al. (2016) [26]	Australia	Qualitative study using semi-structured face-to-face interviews	10 bachelor of Nursing Science students in their final semester of their program at a regional Australian university, purposeful sample	Nursing students	All participants participated in the palliative care curriculum for undergraduates and had received clinical palliative care experiences. However, participant responses were not consistent in reflecting high degrees of self-efficacy in the four documented palliative care capabilities Further educational interventions focused on developing student self-efficacy are necessary to improve graduate nursing student’s belief in their palliative care capabilities	#3
Hermann et al. (2016) [19]	US	Descriptive study	A large public metropolitan university, interprofessional faculty team who developed the iCOPE curriculum	Medical, nursing, social work, and chaplaincy faculty and students	Framework selected to guide curriculum development was based on Clinical Practice Guidelines for Quality Palliative care developed by the National Consensus Project for Quality Palliative Care iCOPE Curriculum components: (1) four online didactic modules, (2) palliative care observational clinical experience, (3) critical reflective writing assignment, and (4) interdisciplinary evidence-based practice simulated case experience Challenges: developing content relevant for multiple disciplines, curriculum placement, scheduling interdisciplinary sessions, representation of students, finding psychometrically sound instruments to measure IPE outcomes; sustainability of funding	#1, #2
Morita et al. (2014) [25]	Japan	Randomized control trial using the waiting list control (nurses who participated in the second education session were the control group)	76 nurses recruited through Japanese palliative care and nursing journals with: (1) three or more years of experience, (2) experience caring for 50 or more terminally ill cancer patients per year, and (3) working at palliative care units/inpatient hospices, in palliative care teams, or general medical wards	Nurses	Two-day, nine-session workshop led by a multidisciplinary team Teaching modalities: lectures, case vignette, small group discussion, and role-plays Program found to have a significant effect on nurses’ level of confidence Strengths of program: structure, delivered by trained facilitators, and existing standardized assessment tools	#1, #3
Nicholl, Price and Tracey (2016) [17]	Ireland	Mixed-methods: quantitative and qualitative	15 students representing practicing professionals in pediatric palliative care delivery	Nurses, social workers, psychotherapists, and chaplains	Modules provided opportunity for interprofessional learning. Program promoted awareness of teamwork required in pediatric palliative care. Face-to-face time important in sharing clinical experiences	#1, #2, #3
Roze des Ordons et al. (2017) [22]	Canada	Longitudinal, evidence-based study	7 critical care medicine fellows in an academic medical center	Physicians (fellows)	Program content informed by literature review and assessment survey results from fellows, attending physician, nurses, and social workers. Curriculum included five 4-h classroom-based sessions. Most helpful instruction methods: instructor-led presentations, simulated practice with actors, observation, debriefing	#1, #3
Schulz et al. (2015) [18]	Germany	Longitudinal, mixed-methods pilot study	University setting, medical student enrolled in palliative education curriculum	Medical students	Students requested additional direct patient contact, opportunities to address personal emotions, and respond to patient needs. Interprofessional, e-learning, and blended-learning approaches were the most valued. Self-efficacy and self-perceived competence regarding care for dying patients improved	#1, #2, #3
Wagner et al. (2013) [24]	Germany	Mixed-methods study	125 healthcare professionals from 35 countries who attended the International Paediatric Palliative Care Course in 2010 and 2012	Physicians, nurses psychologists, social workers, and other healthcare professionals	Course goals: to provide palliative care knowledge and skills, share experience with colleagues, network, and improve multi-professional work. Course included lectures and workshops on challenging cases in pediatric palliative care and clinical observational experiences at a local pediatric palliative care center	#1, #2
Widger et al. (2016) [12]	Canada	Pre–post-test design and integrated knowledge translation approach	Five master facilitators to train 3–5 regional team members at each of the 16 participating pediatric oncology program sites. Regional team members to deliver curriculum to end-users at each of the 16 pediatric oncology programs	Oncology, palliative care, and community home care nurses and community pediatricians	Implement and evaluate national roll-out of the Education in Palliative and End-of-Life Care for Pediatrics (EPEC-Pediatrics) using a ‘Train-the-Trainer’ model. Palliative care QI projects led by regional teams Plan to assess: (1) self-assessed knowledge of health professionals;(2) knowledge dissemination outcomes, (3) practice change outcomes, and (4) quality of palliative care. Patients and families will complete assessments about quality of palliative care	#1, #2, #3
Wittenberg et al. (2014) [16]	US	Pre–post-test design	177 self-selected participants who were continuing education university-based health system account holders or had completed an End-of-Life Nursing Education Consortium course	Nurses, physicians, and other (unidentified) disciplines	End-of-life and disease recurrence were the most challenging conversations. Findings support the value and accessibility of the COMFORT online palliative care communication training and its use in interprofessional palliative care education	#1, #2, #3
Wittenberg et al. (2016) [20]	US	Pre–post-test design	58 interprofessional palliative care team members (29 teams) competitively selected to attend a 2-day training using the COMFORT Communications for Palliative Care Teams curriculum	Nurses, social workers, physicians, chaplains, and psychologist	Moderate communication in participants’ institutions. Bereavement and survivorship care were the weakest areas of communication. Participants of the statewide course taught 962 providers statewide and implemented institution-specific trainings and education materials. Lack of institutional support was the primary barrier	#1, #2, #3

^a^Themes: #1 effective modalities and teaching strategies, #2 interprofessional curriculum, #3 evaluating programs

### Results

Three themes emerged from this integrative literature review: (1) establishing effective modalities and teaching strategies for content delivery, (2) developing an interprofessional palliative care curriculum, and (3) evaluating impact on providers’ self-perceived comfort in delivering palliative care as well as patient and family perceptions of the care received (Table 1).

#### Effective modalities and teaching strategies

Effective modalities included face-to-face and web-based didactic content, in addition to clinical palliative care experiences. Head et al. [15] reported benefits and challenges for each modality, with a combination best meeting the needs and time constraints of students. Wittenberg-Lyles et al. [16] reported about an on-line palliative care curriculum, cautioning against an exclusive on-line curriculum which inhibits in-class face-to-face interaction. Similarly, an in-person format extended classroom discussion and encouraged creative problem solving [17]. Shultz et al. [18] reported that medical students requested additional PC patient contact experiences for practical application.

*“To teach practitioners who are already experienced in the field it is imperative that those involved in teaching have credible relevant expertise in children’s palliative care”* [17].

Only three studies were multi-modal, with web and face-to-face content [12, 18, 19]. All other programs consisted of a single modality and time-limited intervention, taking into account clinical practice demands and constraints [17, 20, 21]. Strategies for content delivery included multidisciplinary lectures from experts, simulation, role-play, group discussions, case-based learning, and on-line modules. Interactive teaching methods were integrated into the educational frameworks, highlighting the importance of communication in clinical practice [17, 18, 21, 22]. Delivery of content in a variety of formats provided opportunity for didactic and experiential learning [18], such as conducting a quality-improvement (QI) project [12]. Collectively, these studies suggested that a multi-modal approach, incorporating a variety of teaching strategies, increased the availability of PC content to healthcare providers.

#### Interprofessional curriculum

National mandates requiring interprofessional PC practice were a driving force for curriculum development [17, 18]. Researchers aligned educational programs with the core competencies outlined in the consensus guidelines [9, 18, 19, 23].

Only one program, designed for practicing professionals, incorporated all core competencies utilizing an internationally validated pediatric curriculum; the Education in Palliative and End-of-Life Care for Pediatrics (EPEC-Pediatrics) [12]. Palliative care education that promoted communication and interprofessional engagement broadened awareness of team members’ roles, and confidence in delivering palliative and end-of-life care [17, 20, 23]. The benefits of international IPE included sharing diverse professional experiences, and improving collaboration amongst disciplines [24]. Only one study focused exclusively on PC interprofessional communication training [20]. Use of the consensus guidelines provided a measure of standardization in palliative care education.

#### Evaluating programs

Program evaluations assessed content delivery and effectiveness in developing self-competency and comfort in the provision of palliative care. Evaluations determined the individual benefit to participants, in addition to the sustainability and adaptability of the curriculum, beyond pilot studies in single institutions [18, 21, 25, 26].

*“All aspects of the program together make it an incredible educational experience. Each step has its own unique purpose and value”* [21].

A validated confidence scale to assess nurses’ confidence in caring for terminally ill cancer patients, before and after a two-day educational workshop, as well as other pre–post-test survey evaluation tools were used [15, 25]. The *End*-*of*-*Life Professional Caregiver Survey* focused on the eight domains of the PC consensus guidelines. A second tool, *the Self*-*Efficacy for Interprofessional Experiential Learning Scale*, measured individuals’ perceptions of engaging in a collaborative interprofessional team environment. A study by Wigder et al. [12] was the only one to evaluate provider, patients, and parent perceptions of PC experience, health-professionals’ knowledge attainment, and dissemination of practice change outcomes at a national level.

### Discussion

The benefits of implementing PC practice within the pediatric oncology population, and the importance of IPE are evident in this review. A notable gap was the limited evaluation of practice changes following completion of PC education. Self-report measures were important to evaluating content, and perceived comfort with PC engagement. However, there was no systematic evaluation of educational intervention impact on care delivery. Although clinicians’ self-reported measures are discussed, there is little focus on evaluating how skills and knowledge directly impact providing holistic care, engaging in critical conversations, and effectively managing symptoms. In accordance with the WHO Framework for Action [13], interprofessional palliative care can be evaluated by: (1) patient and family feedback regarding providers’ palliative care competency, (2) standardized measures that analyze the effect on care delivery, and (3) outcomes of care.

#### Eliciting feedback

Validating patients’ and families’ perspectives in order to best understand the patient experience has been strongly recommended [27]. Family meetings and daily rounding are two informal methods, utilizing routine clinical care for eliciting patient and family perspectives. Individual patient and parent evaluations, focus groups, and technology-based survey evaluations are formal methods for collecting patient and family feedback [28].

#### Standardizing measures

The goal of a PC educational curriculum is to directly impact the quality of patient care [12]. To evaluate this impact, valid and reliable measurements must be utilized [29]. The majority of tools focused on clinician self-reported measures, while there was a lack of patient and caregiver tools evaluating perceptions of PC delivery, with the exception of one study. Psychometrically tested instruments are highly desirable. Lown and McIntosh [27] recommended establishing a shared web-based repository for compassionate collaborative care (CCC) tools. Developing a similar repository for PC instruments and measurements would be an effective way to disseminate and make accessible to pediatric oncology clinicians. In return, this would inform evidence-based clinical practice and spearhead research and a growing body of literature. Measurement tools specific to the pediatric palliative care population could be housed on the National Hospice and Palliative Care Organization website.

#### Evaluating outcomes of care

Evaluation of PC education should extend beyond the direct impact on individual patients to clinicians as well. Potential benefits include patient and family member reported quality-of-life, clinical care outcomes and satisfaction [27]. Potential fiscal impact measures include decreased emergency department visits and re-admissions for symptom management [1, 6]. Tracking value-based outcomes with the support of the National Hospice and Palliative Care Organization could provide a robust evaluation of impact on clinical practice. It has been argued that to fully operationalize and sustain patient and family-centered care in palliative practice settings, compassion and collaboration must be enacted and harmonized [30]. Towards that direction, quality indicators for CCC can guide the planning and implementation of changes at a pediatric hospital, such as patient care conferences, institutional palliative care rounds, establishment of a Compassionate Care Network, and bereavement debriefing [30]. All of these CCC indicators are potentially tied to interprofessional palliative care education within pediatric oncology settings, but further research would be needed to demonstrate associations and effect.

#### Implications

Given that PC should begin as early as a diagnosis of a life-limiting condition is set, the majority of pediatric oncology patients and families could benefit. To this direction, the *Palliative Care and Hospice Education and Training Act of 2017* (H.R. 1676) [31] requests funding provisions to promote interprofessional education and research. Financial support for Palliative Care and Hospice Education Centers, provided through H.R. 1676, would have a direct impact in: (1) increasing the number of interprofessional palliative care faculty in academic and clinical settings; (2) promoting education and research in PC and hospice, and (3) standardizing PC education for patients, families and healthcare professionals.

### Conclusions

Although pediatric oncology clinicians care for patients with life-threatening illnesses, there is great variation in their formal palliative care training. Essential components for successful pediatric palliative care educational programs include: (1) establishing effective modalities and teaching strategies for content delivery, (2) developing an interprofessional palliative care curriculum, and (3) evaluating programs. Despite limited evidence of the effects on pediatric oncology practice, suggested evaluation methods are: (1) eliciting patient and family feedback, (2) standardizing care delivery measures, and (3) evaluating outcomes of care.

### Limitations

To our knowledge, this was the first study addressing palliative care education for pediatric oncology clinicians through an interprofessional lens. Given the on-going debate in Congress, our findings are both timely and relevant to the national health policy agenda. Limitations stem from the lack of high-level evidence available on this topic (no systematic reviews or meta-analysis studies met inclusion criteria). Studies utilized clinician self-report measures and there was no systematic evaluation of educational intervention impact on care delivery. Only one study was multi-modal, with all others consisting of a single modality and time-limited interventions. One program incorporated all core competencies, with only one study utilizing an internationally validated pediatric curriculum. Further longitudinal and multi-site studies are needed to standardize clinician education and develop psychometrically tested tools to establish best practice recommendations.
